# Supplementary material for: Cycles of autoubiquitination and deubiquitination regulate the ERAD ubiquitin ligase Hrd1
Source: eLife. 2019 Nov 12;8:e50903. doi: 10.7554/eLife.50903 (PMC6914336; doi:10.7554/eLife.50903)
Supplement: Supplementary file 1. [file elife-50903-supp1.docx]

Supplementary Table 1. Yeast strains used in this study

| Strain name | Genotype | Source | Figures |
| --- | --- | --- | --- |
| BY4741 | MATa *his3Δ1 leu2Δ0 met15Δ0 ura3Δ0* | GE Dharmacon | 1B, 1E, 1F, 3A, S1D, S2C, S2D |
| *hrd1Δ* | MATa *his3Δ1 leu2Δ0 met15Δ0 ura3Δ0 hrd1::kanR* | GE Dharmacon | 1A, 1B, 3B, S1A, S5A-S5D |
| *hrd3Δ* | MATa *his3Δ1 leu2Δ0 met15Δ0 ura3Δ0 hrd3::kanR* | GE Dharmacon | 1B-1F, 2, 3E, 4B, S1B, S2C, S4, S5A |
| *usa1Δ* | MATa *his3Δ1 leu2Δ0 met15Δ0 ura3Δ0 usa1::kanR* | GE Dharmacon | 1B, 3C, S1C, S2A |
| *der1Δ* | MATa *his3Δ1 leu2Δ0 met15Δ0 ura3Δ0 der1::kanR* | GE Dharmacon | 1B, 3D, S2B |
| *ubp1Δ* | MATa *his3Δ1 leu2Δ0 met15Δ0 ura3Δ0 ubp1::kanR* | This study | 1E, 1G, 1H |
| *hrd1Δhrd3Δ*  (yRB0057A) | MATa *his3Δ1 leu2Δ0 MET15 lys2Δ0 ura3Δ0 hrd1::kanR hrd3::kanR* | This study | 3F-3N |
| *hrd1Δubp1Δ*  (yBGP15A) | MATa *his3Δ1 leu2Δ0 met15Δ0 ura3Δ0 hrd1::kanR ubp1::kanR* | This study | S3A-D, 2D |
| *hrd3Δusa1Δ*  (yRB0065A) | MATa *his3Δ1 leu2Δ0 met15Δ0 ura3Δ0 hrd3::kanR usa1::kanR* | This study | 4A, S5A |
| *hrd3Δubp1Δ*  (yRB0126) | MATa *his3Δ1 leu2Δ0 met15Δ0 ura3Δ0 hrd3::kanR ubp1::hphNT1* | This study | 1E |
| *hrd3Δubp1Δusa1Δ*  (yRB0075C) | MAT? *his3Δ1 leu2Δ0 MET15 ura3Δ0 hrd3::kanR usa1::kanR ubp1::hphNT1* | This study | 4C, 4D |
