# Supplementary material for: Cycles of autoubiquitination and deubiquitination regulate the ERAD ubiquitin ligase Hrd1
Source: eLife. 2019 Nov 12;8:e50903. doi: 10.7554/eLife.50903 (PMC6914336; doi:10.7554/eLife.50903)
Supplement: Supplementary file 2. [file elife-50903-supp2.docx]

**Supplementary Table 2. Plasmids used in this study**

| Plasmid name | Backbone, features |  | Reference | Figures |
| --- | --- | --- | --- | --- |
| pRS313 | *HIS3*, centromeric |  | (Sikorski and Hieter, 1989) | S3A-C, S5B-S5D |
| pRS315 | *LEU2*, centromeric |  | (Sikorski and Hieter, 1989) | 4B, S3B-C, S5C, S5D |
| pRS416 | *URA3*, centromeric |  | (Sikorski and Hieter, 1989) | 1B-1D, 1F, 1H, 2B-2D, 3, 4A, 4C, S2A-S2C, S2E, S3, S4, S5B-S5D |
| pRB108 | pRS313_Hrd1 |  | (Baldridge and Rapoport, 2016) | 3G, 3L, S4B-S5D |
| pRB264 | pRS313_Hrd1_KRK |  | (Baldridge and Rapoport, 2016) | 3I, 3M |
| pRB092 | pRS313_Hrd1_C399S |  | (Baldridge and Rapoport, 2016) | 3K, 3N |
| pRB408 | pRS313_Hrd1_His10 |  | (Baldridge and Rapoport, 2016) | 1F, 1H, S2C |
| pRB414 | pRS416_Hrd3 |  | This study | 4A, 4C |
| pRB450 | pRS313_Hrd1_3xFlag |  | (Baldridge and Rapoport, 2016) | 1A-1C, 1E, 1G, 2B-2E, 3A-3F, 4A-4C, S1A, S2A, S2B, S2E, S3, S4A-D, S5A |
| pRB437 | pRS313_Hrd1_KRK_3xFlag |  | (Baldridge and Rapoport, 2016) | 3H |
| pRB454 | pRS313_Hrd1_C399S_3xFlag |  | (Baldridge and Rapoport, 2016) | 1D, 3J |
| pRB640 | pRS315_CPY*_3xFlag |  | This study | 3G, 3I, 3K, S1D |
| pRB751 | pRS416_Erg3_3xFlag |  | This study | 3L-3N, S3C |
| pRB415 | pRS413_3xFlag_Hrd3 | Internal flag tag, inserted between amino acids 20 and 21 | This study | S1B |
| pRB416 | pRS313_3xFlag_Usa1 |  | This study | S1C |
| pRB600 | pRS416_GPD_3xHA |  | This study | 1A, S1A-S1D |
| pRB601 | pRS416_GPD_3xHA_YUH1 |  | This study | 1A, S1A-S1D |
| pRB602 | pRS416_GPD_3xHA_UBP1 |  | This study | 1A, S1A-S1D |
| pRB603 | pRS416_GPD_3xHA_UBP2 |  | This study | 1A, S1A-S1D |
| pRB604 | pRS416_GPD_3xHA_UBP3 |  | This study | 1A, S1A-S1D |
| pRB605 | pRS416_GPD_3xHA_UBP5 |  | This study | 1A, S1A-S1D |
| pRB606 | pRS416_GPD_3xHA_UBP6 |  | This study | 1A, S1A-S1D |
| pRB607 | pRS416_GPD_3xHA_UBP7 |  | This study | 1A, S1A-S1D |
| pRB608 | pRS416_GPD_3xHA_UBP8 |  | This study | 1A, S1A-S1D |
| pRB609 | pRS416_GPD_3xHA_UBP9 |  | This study | 1A, S1A-S1D |
| pRB610 | pRS416_GPD_3xHA_UBP10 |  | This study | 1A, S1A-S1D |
| pRB611 | pRS416_GPD_3xHA_UBP11 |  | This study | 1A, S1A-S1D |
| pRB612 | pRS416_GPD_3xHA_UBP12 |  | This study | 1A, S1A-S1D |
| pRB613 | pRS416_GPD_3xHA_UBP13 |  | This study | 1A, S1A-S1D |
| pRB614 | pRS416_GPD_3xHA_UBP14 |  | This study | 1A, S1A-S1D |
| pRB615 | pRS416_GPD_3xHA_UBP15 |  | This study | 1A, S1A-S1D |
| pRB616 | pRS416_GPD_3xHA_UBP16 |  | This study | 1A, S1A-S1D |
| pRB617 | pRS416_GPD_3xHA_DOA4 |  | This study | 1A, S1A-S1D |
| pRB618 | pRS416_GPD_3xHA_SAD1 |  | This study | 1A, S1A-S1D |
| pRB619 | pRS416_GPD_3xHA_OTU1 |  | This study | 1A, S1A-S1D |
| pRB620 | pRS416_GPD_3xHA_OTU2 |  | This study | 1A, S1A-S1D |
| pRB621 | pRS416_GPD_3xHA_RRI1 |  | This study | 1A, S1A-S1D |
| pRB622 | pRS416_GPD_3xHA_RPN11 |  | This study | 1A, S1A-S1D |
| pRB624 | pRS416_GPD_3xHA_RPN8 |  | This study | 1A, S1A-S1D |
| pRB631 | pRS416_GPD_Ubp1 |  | This study | 1B-1H, 3, 4A-4C, S2A-S2C, S4B, S5B |
| pRB635 | pRS416_GPD_Ubp1_C110S |  | This study | 1B-1D, 4A, 4C, S2A, S2B, S5B |
| pRB659 | pRS416_GPD_Ubp1_3xHA |  | This study | 1G, 2B-2D, S4A |
| pRB660 | pRS416_GPD_Ubp1_C110S_3xHA |  | This study | 2B, S4A |
| pRB662 | pRS416_GPD_Ubp1_ΔTM_3xHA | Deletion of first 66 amino acids | This study | 2B, S4B |
| pRB779 | pRS416_Ubp1_3xHA | Endogenous promoter | This study | 1G, S2E, S4B |
| pRB780 | pRS416_Ubp1_C110S_3xHA | Endogenous promoter | This study | S4A |
| pRB734 | pRS416_GPD_TM(Cue4)_Ubp1_3xHA | Amino acids 5-22 of Cue4 replacing amino acids 34-51 of Ubp1 | This study | 2C, S4A |
| pRB735 | pRS416_GPD_Nterm(Cue4)_Ubp1_3xHA | Amino acids 1-23 of Cue4 replacing amino acids 1-67 of Ubp1 | This study | 2C, S4A |
| pRB737 | pRS416_GPD_ Hdomain(Usa1)_Ubp1_3xHA | H domain is amino acids 437-490 from Usa1 replacing amino acids 1-96 in Ubp1 | This study | 2C, S4A |
| pRB766 | pRS416_GPD_TM(Cue4)_Hdomain(Usa1)_3XHA | H domain is amino acids 437-490 from Usa1 | This study | S4B |
| pRB781 | pRS416_GPD_TM(Ubp1)_hUSP2_3xHA | Amino acids 1-100 of Ubp1 fused to the USP domain of hUSP2 (amino acids 267-605) | This study | 2D, S4A |
| pRB782 | pRS416_GPD_Hdomain(Usa1)_hUSP2_3xHA | H domain is amino acids 437-490 from Usa1 fused to the USP domain of hUSP2 (amino acids 267-605) | This study | 2D, S4A |
| pRB783 | pRS416_GPD_hUSP2_3xHA | hUSP2 (amino acids 267-605) | This study | 2D, S4A |
| pRB428 | pRS416_GPD_Usa1 |  | This study | 4A, 4C, S5B |
| pRB429 | pRS416_GPD_Usa1_ΔUBL | (Δ260-317) | This study | 4A, 4C, S5B |
| pRB669 | pRS415_GPD_Usa1_ΔUBL | (Δ260-317) | This study | 4B, 4D, S5C, S5D |
| pRB670 | pRS415_GPD_Usa1 |  | This study | 4B, 4D, S5C, S5D |
| pRB798 | pRS416_Ubp1_3xV5 |  | This study | 1G, 2D, S2E, S3A, S3D |
| pRB799 | pRS416_GPD_Ubp1_3xV5 |  | This study | S3A, S3D |
| pBGP1 | pRS416_GPD_Hmg2_myc_NR1_GFP |  | This study | S5C |
| pBGP3 | pRS416_GPD_Hmg2_GFP |  | This study | S3B, S5D |
| pBGP134 | pBGP134_pRS415_GPD_ss-GFPfast-Cpy*-HDEL_pgk1 |  | This study | S3A |
| pBGP287 | pRS416_Ubp1_TMCue4_3xV5 |  | This Study | 2D |
| pBGP290 | pRS416_Ubp1_Δ66_3xV5 |  | This study | 2D |
| pBGP291 | pRS416_Ubp1_Hdomainprebox1_3xV5 |  | This study | 2D |
| pBGP292 | pRS415_Ubp1 |  | This study | S3B-C |
| pRB296 | pRS315_CPY*_3xHA |  | (Baldridge and Rapoport, 2016) | 1H, S3D, S5B |
| pRB666 | pRS416_Ubp1 |  | This study | 1H, 4D |
| pBGP170 | pRS415_Orm2_3xFlag |  | This study | 1G, 2E, S2E |
| pRB667 | pRS415_GPD_Ubp1 |  | This study | S5C, S5D |
| pRB668 | pRS415_GPD_Ubp1_C110S |  | This study | S5C, S5D |
